# A novel multiparametric imaging approach to acute myocarditis using T2-mapping and CMR feature tracking

**DOI:** 10.1186/s12968-017-0387-x

**Published:** 2017-09-21

**Authors:** Bettina Baeßler, Melanie Treutlein, Frank Schaarschmidt, Christian Stehning, Bernhard Schnackenburg, Guido Michels, David Maintz, Alexander C. Bunck

**Affiliations:** 1 Department of Radiology, University Hospital of Cologne, Kerpener Str. 62, D-50937 Cologne, Germany; 2 Institute of Biostatistics, Faculty of Natural Sciences, Leibniz Universität Hannover, Hannover, Germany; 3 Philips Research, Hamburg, Germany; 4 Philips, Healthcare Germany, Hamburg, Germany; 5 Department III of Internal Medicine, Heart Centre, University Hospital of Cologne, Cologne, Germany

**Keywords:** Cardiac magnetic resonance imaging, Strain, Strain rate, Myocardial inflammation, Myocarditis, T2-mapping, Feature tracking

## Abstract

**Background:**

The aim of this study was to evaluate the diagnostic potential of a novel cardiovascular magnetic resonance (CMR) based multiparametric imaging approach in suspected myocarditis and to compare it to traditional Lake Louise criteria (LLC).

**Methods:**

CMR data from 67 patients with suspected acute myocarditis were retrospectively analyzed. Seventeen age- and gender-matched healthy subjects served as control. T2-mapping data were acquired using a Gradient-Spin-Echo T2-mapping sequence in short-axis orientation. T2-maps were segmented according to the 16-segments AHA-model and segmental T2 values and pixel-standard deviation (SD) were recorded. Afterwards, the parameters maxT2 (the highest segmental T2 value) and madSD (the mean absolute deviation (MAD) of the pixel-SDs) were calculated for each subject. Cine sequences in three long axes and a stack of short-axis views covering the left and right ventricle were analyzed using a dedicated feature tracking algorithm.

**Results:**

A multiparametric imaging model containing madSD and LV global circumferential strain (GCS_LV_) resulted in the highest diagnostic performance in receiver operating curve analyses (area under the curve [AUC] 0.84) when compared to any model containing a single imaging parameter or to LLC (AUC 0.79). Adding late gadolinium enhancement (LGE) to the model resulted in a further increased diagnostic performance (AUC 0.93) and yielded the highest diagnostic sensitivity of 97% and specificity of 77%.

**Conclusions:**

A multiparametric CMR imaging model including the novel T2-mapping derived parameter madSD, the feature tracking derived strain parameter GCS_LV_ and LGE yields superior diagnostic sensitivity in suspected acute myocarditis when compared to any imaging parameter alone and to LLC.

## Background

Due to the heterogeneity of clinical presentations, the diagnosis of acute myocarditis remains one of the most challenging in cardiology [1]. Nevertheless, a timely and correct diagnosis is important for a tailored therapeutic strategy in order to reduce the risk of progression to chronic active disease and/or dilated cardiomyopathy [2, 3].

The current gold standard, endomyocardial biopsy (EMB) is limited by its periprocedural risks and the low diagnostic sensitivity due to the so-called sampling error [1, 4]. As a consequence, cardiovascular magnetic resonance (CMR) has now become the reference non-invasive diagnostic tool in suspected myocarditis [5]. Due to its unique capability of combining morphological and functional imaging with myocardial tissue characterization, CMR enables the detection of the typical features of acute inflammation, such as myocardial dysfunction, edema, hyperemia, and necrosis.

However, the current “Lake Louise Criteria” (LLC) [5] for CMR-based diagnosis of myocarditis, i.e. T2-weighted edema imaging, early gadolinium enhancement (EGE) and late gadolinium enhancement (LGE) still lack diagnostic accuracy [6, 7]. Recently, novel quantitative CMR techniques such as T1 and T2 mapping and feature tracking (FT) based strain analysis have emerged as potential novel diagnostic tools, aiming at an improved diagnostic accuracy in suspected myocarditis [7–14].

Myocardial strain parameters thereby represent new quantitative indices of cardiac deformation and are thought to be more sensitive markers of contractile dysfunction than left ventricular ejection fraction (LVEF). A decline in strain values has been shown to precede decreases in LVEF in many different diseases [15], including acute myocarditis [8, 16].

Mapping techniques yield absolute T1 and T2 relaxation times and thus offer a quantitative assessment of focal, but also diffuse myocardial tissue alterations. Their routine clinical use, however, is still hindered by one major limitation, i.e. the large overlap of average T1 and T2 times between healthy individuals and myocarditis patients [17–19], leading to difficulties in discriminating “normal” and “injured” myocardium when averaging T1 or T2 values over the myocardium. In order to face these challenges, our group recently proposed a novel approach to T2 mapping [17]. Using the novel quantitative T2 mapping-derived parameters maxT2 and madSD, which aimed at a better reflection of the tissue inhomogeneity, i.e. the spatial variation of myocardial inflammation in suspected myocarditis, resulted in a similar diagnostic performance compared to LLC in a moderately large confirmatory study in 97 patients [20].

A small set of recent studies suggested that the future of diagnosing myocarditis will most likely be a multiparametric imaging approach combining several of the novel quantitative parameters within a single imaging protocol [7, 11, 13, 21]. The aim of the present study therefore was to integrate the novel T2 mapping approach as well as FT derived myocardial strain parameters in a multiparametric imaging protocol and to test, whether this approach would result in a further increase in diagnostic potential to detect suspected acute myocarditis with preserved LVEF when compared to single diagnostic parameters and to LLC.

## Methods

### Study population

After obtaining approval by the local institutional review board, data sets of 86 patients who had been consecutively referred to our department for CMR imaging after clinical diagnosis of acute myocarditis (mean symptom duration before referral: 4.8 ± 4.4 days; all patients demonstrated symptoms for less than 14 days) were retrospectively analyzed. The clinical diagnosis of myocarditis was based upon the clinical suspicion of acute myocarditis and on the clinical criteria in the current recommendations [22] (Table 1). Patients with CMR findings characteristic for other diseases than myocarditis were excluded from further analyses. Of 86 available data sets, 4 were excluded due to severe respiratory motion artifacts in T2 mapping data resulting in non-diagnostic image quality. 2 patients had to be excluded because of lacking T2 black blood (T2 BB), EGE and LGE imaging and 4 datasets were excluded from further analyses due to severe motion artifacts in cine imaging not suitable for subsequent FT analyses. Finally, datasets of 76 patients with suspected myocarditis were analyzed in a retrospective fashion. Of these 76 datasets, only patients with preserved LVEF (defined as ≥ 50%) were included in further statistical analyses (*n* = 67).

**Table 1:** Classification of patients with suspected myocarditis according to clinical criteria [22]

	Myocarditis patients (*n* = 67)
Clinical symptoms consistent with myocarditis [%]	100
Acute chest pain	71
New-onset (days up to 3 months) or worsening of: dyspnea at rest or exercise / fatigue, with or without left and/or right heart failure signs	40
Palpitations / arrhythmia symptoms / syncope / aborted sudden cardiac death	12
Cardiogenic shock	2
Diagnostic criteria consistent with myocarditis [%]	100
ECG / Holter / stress test features	82
Elevated TnT/TnI	55
Functional and structural abnormalities on cardiac imaging (echo/angio/CMR)	32
Exclusion of coronary artery disease (CAD) [%]	100
Cardiac catheterization	51
Cardiac computed tomography angiography	41
Clinically (young patients)	8

CMR data from 17 age and gender matched healthy volunteers served as control. Healthy subjects were selected as previously described [23], inclusion criteria being: i) uneventful medical history, ii) no symptoms of inflammation, iii) absence of any symptoms indicating cardiovascular dysfunction, iv) normal cardiac dimensions and function on cine CMR. For each volunteer written informed consent was obtained prior to the study after approval by the local institutional review board.

Characteristics of patients and controls are shown in Table 2.

**Table 2:** Characteristics of patients with suspected myocarditis and controls

Parameter	Controls	Myocarditis patients	*p*-value
Number	17	67	n.a.
Females / Males	6/11	18/49	n.a.
Age [years]	36 ± 12	37 ± 14	.849
Height [cm]	178 ± 11	177 ± 9	.788
Weight [kg]	77 ± 14	79 ± 14	.671
Heart rate [bpm]	63 ± 15	65 ± 13	.743
Symptom duration before CMR [days]	n.a.	4.8 ± 4.4	n.a.
Initial TnT [μg/l]	n.a.	3.0 ± 16.1	n.a.
Initial NT-proBNP [pg/ml]	n.a.	2380 ± 5535	n.a.
Initial CK [U/l]	n.a.	2942 ± 1719	n.a.
Initial CRP [mg/l]	n.a.	37.4 ± 43.5	n.a.
LV^a^ ED^b^ volume / BSA^c^ [ml/m^2^]	81 ± 14	84 ± 21	.486
LV ES^d^ volume / BSA [ml/m^2^]	29 ± 8	33 ± 10	.081
LV ejection fraction [%]	65 ± 5	62 ± 7	**.032**
LV ED wall mass / BSA [g/m^2^] (without papillary muscles)	47 ± 11	54 ± 15	**.026**
T2-Ratio	1.8 ± 1.4	2.2 ± 0.9	.097
≥ 2 out of 3 LLC^e^ [%]	0	57	n.a.
1 out of 3 LLC [%]	24	27	n.a.
0 out of 3 LLC [%]	76	16	n.a.

^a^ *LV* left ventricle, ^b^ *ED* end diastolic, ^c^ *BSA* body surface area, ^d^ *ES* end systolic, ^e^ *LLC* Lake Louise criteria

Statistical significant *p* values are printed in bold face

### CMR examination

CMR was performed on a 1.5 T MR system (Achieva, Philips Healthcare, Best, The Netherlands) using a standard five-element cardiac phased array coil and a 4-lead vectorcardiogram. A balanced steady-state free precession (b-SSFP) sequence in breath-hold technique and with retrospective ECG-triggering was acquired in three horizontal long axes and a stack of short axes (SAX) covering the left ventricle (LV) for functional analysis and subsequent Feature Tracking analyses. Imaging parameters were chosen as previously described [23]. Volumetry was performed on a standard post-processing platform (IntelliSpace Portal, Version 6, Philips Healthcare).

In patients and controls, edema-sensitive black blood T2-weighted images with fat saturation were acquired in SAX orientation covering the entire LV [24]. Myocardial early gadolinium enhancement was assessed in all patients using fast spin- echo T1-weighted images in axial orientation during the first minutes after 0.1 mmol/kg Gd-DOTA (Dotarem; Guerbet, Villepinte, France) contrast administration as previously described [25]. LGE imaging was performed in all patients 15 min after 0.2 mmol/kg Gd-DOTA (Dotarem; Guerbet, Villepinte, France) contrast administration using an inversion-recovery gradient-echo sequence in the horizontal long axes and SAX as previously described [26].

T2-mapping data were acquired in 3 SAX slices evenly distributed across the LV (resulting in one apical, midventricular and basal slice) using an ECG-triggered, breathhold Gradient Spin Echo technique (GraSE) [27]. Image parameters were chosen as previously described [17]. A pixel-wise myocardial T2-map was generated using a mono-exponential fit [9] on the magnitude data using a maximum likelihood estimator (MLE), where a Rician noise distribution was assumed.

### CMR image analysis

#### Lake Louise criteria

Image analysis of LLC was performed using a standard post-processing platform (Intelli Space Portal, Version 6, Philips Healthcare). The myocardium was evaluated for presence of myocardial edema on T2-weighted black-blood imaging visually as well as by calculating the T2-ratio (deemed pathological when ≥1.9) as previously described [5, 28]. For calculation of the early gadolinium rnhancement ratio (EGEr), standardized myocardial and skeletal muscle regions of interest (ROIs) were drawn on one axial slice before and after contrast administration. EGEr was calculated as previously described and deemed pathological when ≥4 [29]. The myocardium was visually assessed on LGE images and considered suspicious for myocarditis in cases of focal signal intensity alterations with a subepicardial or intramyocardial pattern typical for myocarditis [5]. CMR diagnosis of myocarditis was based upon the presence of ≥2 out of 3 LLC [5].

#### T2-mapping

All analyses were blinded with respect to the status “healthy” or “myocarditis” of each individuum. T2-maps were calculated with a dedicated plug-in written for the OsiriX viewer for Mac OS X (version 5.8.5, Pixmeo, Bernex, Switzerland) as previously described [23]. The myocardial ROI was automatically segmented according to the 16-segments AHA-model [30] and T2 values were calculated for each segment. While averaging all pixels within one myocardial segment for segmental T2 calculation, the corresponding standard deviation was recorded and assigned to the additional parameter “pixel-SD” as previously described [17].

Two statistically derived parameters of myocardial T2 and pixel-SD were calculated, aiming at reflecting the increased tissue inhomogeneity in the case of myocardial inflammation [17]: a) the maximum segmental T2 value (maxT2; defined by the one segment of all 16 segments exhibiting the highest segmental T2 value), and b) the mean absolute deviation (MAD) of segmental pixel-SD (madSD).

#### CMR feature tracking (FT) based strain analysis

FT was performed offline based on the acquired bSSFP cine images and using a dedicated software (Image-Arena VA Version 3.0 and 2D Cardiac Performance Analysis MR Version 1.1.0; TOMTEC Imaging Systems, Unterschleissheim, Germany). All analyses were blinded with respect to the status “healthy” or “myocarditis” of each individuum. Endocardial contours were drawn manually in end-diastolic images with subsequent software-driven automatic tracking of the endocardial contour throughout the entire cardiac cycle. The quality of automatic tracking was checked and contours were manually adjusted and tracking repeated were deemed necessary. The four-chamber view was used to derive right ventricular (RV) and LV longitudinal strain and strain rate (SR) values. Circumferential strain and SR parameters of both RV and LV were determined in short axis view at a basal, midventricular and apical level of the ventricle as previously described [8]. Afterwards, values were averaged over all three SAX slices in order to obtain global strain and SR parameters.

### Statistical analysis

Statistical analysis was performed in R 3.1.2 [31], using the packages ggplot2 [32] for graphical visualization, pastecs [33] for descriptive statistics, rpart [34] for fitting single classification trees [35], and ROCR [36] for using receiver operating curve (ROC) analyses.

All continuous data are given as mean ± standard deviation. A *p*-value of < .05 was regarded as statistically significant. Testing for significant differences between patients and controls was performed using Wilcoxon sum rank test or Welch independent T-test, depending on normality distribution of the data. Classification models were built using multiple logistic regression analyses. Models were compared according to the Akaike Information Criterion (AIC; where lower AIC represents a better model fit). Optimal cut-off values were defined using classification trees as previously described [17]. The diagnostic accuracy as well as diagnostic sensitivity and specificity of optimal predictive parameters were evaluated using ROC analyses. In models with two (three) parameters, sensitivity was computed as the proportion of myocarditis patients with 2 (3) out of 2 (3) parameters exceeding the cut-off, relative to all myocarditis patients.

## Results

### Feature tracking derived myocardial strain parameters in acute myocarditis

**Fig. 1:**
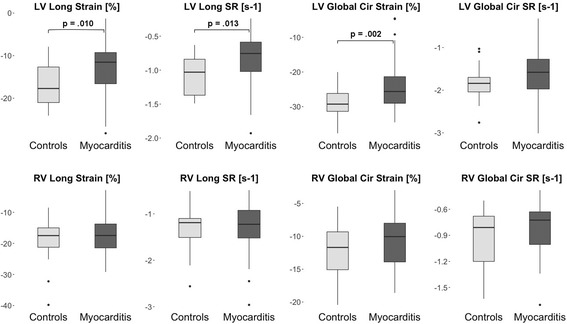
Box-Whisker plots representing the differences of LV and RV strain parameters between controls and patients with suspected myocarditis. The centreline in each box represents the median, whereas the lower and upper limits of each box represent the 25th and 75th percentiles, respectively. Whiskers extend to the most extreme observations within 25th and 75th percentiles ±1.5*IQR. Observations outside these whiskers are shown as dots. LV - left ventricle; RV - right ventricle; IQR - inter-quartile-range; SR - strain rate

Significant differences between patients with suspected myocarditis and controls were observed for LV global longitudinal strain (GLS_LV_) and SR, and LV global circumferential strain (GCS_LV_; Fig. 1; Table 3). No significant differences were observed for any of the RV parameters (Fig. 1; Table 3). All strain parameters revealed a wide range of values in controls and patients as well as a considerable overlap between the values of the two groups (Fig. 1).

**Table 3:** Myocardial strain and T2 parameters of controls and patients with suspected myocarditis

Parameter	Controls	Myocarditis patients	*p*-value
LV^a^ longitudinal strain (GLS_LV_) [%]	−17 ± 5	−14 ± 6	**.032**
LV longitudinal SR^b^ (s^−1^)	−1.1 ± 0.3	−0.9 ± 0.4	**.049**
LV circumferential strain (GCS_LV_) [%]	−29 ± 4	−26 ± 4	**.014**
LV circumferential SR (s^−1^)	−1.9 ± 0.4	−1.7 ± 0.5	.362
RV^c^ longitudinal strain [%]	−20 ± 8	−18 ± 7	.488
RV longitudinal SR (s^−1^)	−1.4 ± 0.5	−1.4 ± 6	.952
RV circumferential strain [%]	−12 ± 4	−12 ± 4	.814
RV circumferential SR (s^−1^)	−0.9 ± 0.4	−0.9 ± 0.3	.976
Mean T2 [ms]	58 ± 5	63 ± 6	**< .001**
maxT2 [ms]	69 ± 11	79 ± 13	**.002**
Mean SD [ms]	7.9 ± 2.3	9.9 ± 3.1	**.006**
madSD^d^ [ms]	1.7 ± 1.0	2.9 ± 1.4	**< .001**

^a^ *LV* left ventricle, ^b^ *SR* strain rate, ^c^ *RV* right ventricle, ^d^ *madSD* mean absolute deviation of pixel-SD

Statistical significant *p* values are printed in bold face

**Fig. 2:**
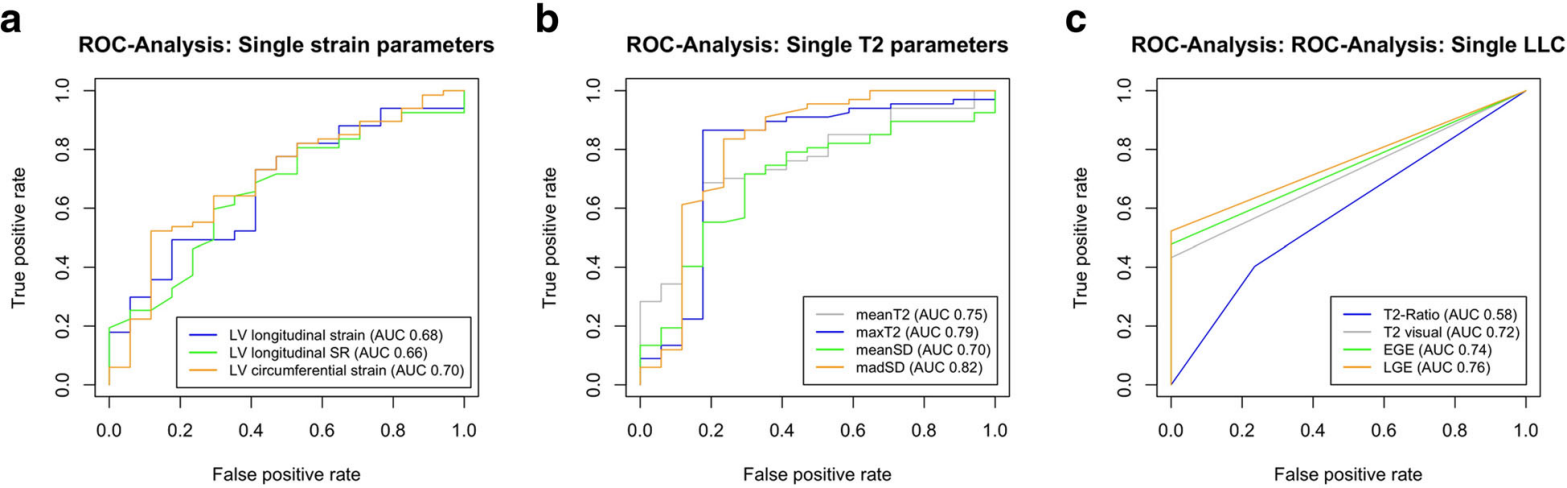
ROC-Analysis for selected LV strain parameters (**a**), single T2 parameters (**b**), and single LLC (**c**) in order to differentiate patients with suspected myocarditis from controls. LLC - Lake Louise criteria, LV - left ventricle

Among several multiple logistic regression models including different strain parameters, GCS_LV_ performed best as an independent predictor of suspected myocarditis according to the AIC (81.69). When using different strain parameters in combination, all models revealed collinearity of the selected strain parameters, as either one was only significant in the model when the other one was not included. ROC-analyses finally revealed an only moderate potential in differentiating healthy controls from myocarditis patients for the three LV strain parameters (Fig. 2a; Table 4).

**Table 4:** Diagnostic performance of different CMR parameters for diagnosing suspected acute myocarditis

Parameter	AUC^a^	Sensitivity [%]	Specificity [%]	PPV^b^ [%]	NPV^c^ [%]	Accuracy [%]
Lake Louise criteria						
T2-Ratio (≥1.9)	0.58	40	77	87	25	48
Visual edema	0.72	43	100	100	31	55
EGEr^d^	0.74	48	100	100	33	58
LGE^e^	0.76	52	100	100	35	62
Strain parameters						
LV^f^ longitudinal strain	0.68	73	59	88	36	70
LV longitudinal SR^g^	0.66	60	71	89	31	62
LV circumferential strain	0.70	52	88	95	32	60
T2 parameters						
Mean T2	0.75	69	82	94	40	71
MaxT2	0.79	87	82	95	61	86
Mean SD	0.70	72	71	91	39	71
MadSD	0.82	84	77	93	54	82
Multiparametric models *without* gadolinium						
madSD + LV longitudinal strain	0.84	93	71	93	71	88
madSD + LV circumferential strain	0.84	93	82	95	74	91
madSD ≥1.8 ms + LV circumferential strain ≥ −25%	0.84	90	77	95	67	88
Multiparametric models *with* gadolinium						
LLC^h^	0.79	58	100	100	38	67
madSD + LV longitudinal strain + LGE	0.94	99	77	94	93	94
madSD + LV circumferential strain + LGE	0.93	97	82	96	88	94
madSD ≥1.8 ms + LV circumferential strain ≥ −25% + LGE pos.	0.94	97	77	96	88	94

^a^ *AUC* area under the curve, ^b^ *PPV* positive predictive value, ^c^ *NPV* negative predictive value, ^d^ *EGEr* Early Gadolinium Enhancement ratio, ^e^ *LGE* Late Gadolinium Enhancement, ^f^ *LV* left ventricle, ^g^SR strain rate, ^h^ *LLC* Lake Louise criteria

### T2 mapping derived myocardial tissue parameters in acute myocarditis

**Fig. 3:**
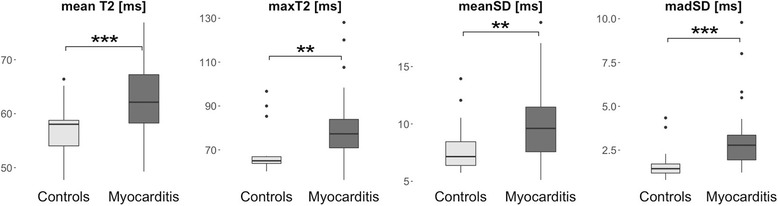
Box-Whisker plots representing the differences of T2 parameters between controls and patients with suspected myocarditis. For detailed description please refer to Fig. 1

All parameters derived from T2 mapping showed significantly higher values in patients with suspected myocarditis when compared to controls (Fig. 3, Table 3). Similar to the strain parameters, however, meanT2 and meanSD both demonstrated a large variation of values in myocarditis patients as well as a considerable overlap between both groups (Fig. 3).

Among several multiple logistic regression models including different T2 parameters alone or in combination, the model containing madSD as an independent predictor of suspected myocarditis performed best according to the AIC (72.04). The model combining madSD and maxT2 as previously recommended [20] resulted in a minimally higher AIC (73.08) and revealed some collinearity between the two parameters, as either one was only significant in the model when the other one was not included.

In ROC analyses, madSD resulted in the highest area under the curve (AUC) when compared to the other three T2 parameters (Fig. 2b, Table 4) and all single T2 parameters were superior to the single strain parameters.

### Lake Louise criteria

All single LLC demonstrated only a moderate potential for discriminating between patients with suspected myocarditis and healthy controls (Fig. 2c; Table 4). A visual analysis of T2 BB edema imaging was superior to T2-ratio in our cohort.

### Multiparametric imaging models for diagnosing acute myocarditis

Finally, different multiparametric imaging models were tested in a sequential fashion in order to evaluate, which combination exhibits the highest diagnostic potential in suspected acute myocarditis.

Multiple logistic regression analyses showed that models containing madSD and either GLS_LV_ or GCS_LV_ performed better than the model containing madSD alone (AIC 66.55 for madSD + GLS_LV_ and 68.12 for madSD + GCS_LV_).

**Fig. 4:**
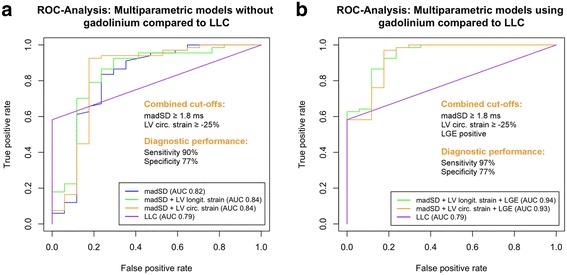
ROC-Analysis for multiparametric imaging models without gadolinium (**a**), and using gadolinium (**b**) compared to LLC in order to differentiate patients with suspected myocarditis from controls. LLC - Lake Louise criteria, LV - left ventricle

In ROC analyses, the multiparametric model containing madSD and GCS_LV_ resulted in the highest diagnostic performance (Fig. 4a; Table 4). Including GLS_LV_ instead of GCS_LV_ in the model resulted in an equal sensitivity but considerably lower specificity (Fig. 4a; Table 4). Both multiparametric models exhibited a slightly better diagnostic performance when compared to the model containing madSD alone and all three models were superior to the LLC (Fig. 4a; Table 4).

Adding LGE to both multiparametric imaging models resulted in further increased diagnostic performances (Fig. 4b, Table 4) and the model containing madSD, GCS_LV_ and LGE yielded the highest diagnostic sensitivity.

### Definition of cut-off values for multiparametric imaging

Using classification trees in order to define optimal cut-off values for the best multiparametric imaging model (madSD + GCS_LV_ + LGE) resulted in a cut-off of ≥1.8 ms for madSD, of ≥ −25% for GCS_LV_ and LGE positive. Using these cut-offs in combination led to an excellent diagnostic performance (Table 4). Using the two cut-offs for madSD and GCS_LV_ without gadolinium (LGE) led to a still excellent discriminatory performance (Table 4).

## Discussion

The present study could demonstrate for the first time that using the novel T2 mapping derived parameter madSD in combination with FT derived global myocardial strain parameters, i.e. GCS_LV_ or GLS_LV_ in a multiparametric imaging approach results in an increased diagnostic performance when compared to any single CMR parameter or to LLC, thereby also highlighting the diagnostic value of madSD in the CMR based diagnostic algorithm of suspected acute myocarditis. The best “native” multiparametric model in our study, madSD + GCS_LV_, increased diagnostic accuracy from 67% for LLC up to 91%, leading to a detection of 23 patients with suspected myocarditis who would have been missed by LLC. Using a multiparametric model including gadolinium-based LGE imaging finally led to a further moderate increase in diagnostic performance with an excellent accuracy of 94%, leading to a detection of even 3 more patients (*n* = 26) who would have been missed by LLC. Finally, this study is the first offering dedicated cut-off values for all diagnostic parameters included in the multiparametric imaging models (Fig. 4).

### Feature tracking derived strain parameters

In the present study, GLS_LV_ and GCS_LV_ showed significant differences of mean values, but also revealed a considerable overlap between healthy subjects and patients with suspected myocarditis, what led to an only moderate diagnostic potential of these two strain parameters when used as single diagnostic parameters in myocarditis patients with preserved LVEF. These observations are in concordance with a previous study of our group focusing on biventricular strain parameters in a different small cohort of myocarditis patients [8] as well as with a recent study by André et al. In these studies, both LV strain parameters were significantly correlated with LVEF, elucidating, that larger differences of mean values between patients and controls might most likely be introduced by patient subgroups with impaired LVEF. Hence, as strain parameters are sought to be more sensitive diagnostic markers than LVEF and thereby might reflect different aspects of LV systolic function [37], we now focused on myocarditis patients with preserved LVEF only. This also might explain the lower diagnostic performance of GLS_LV_ in our study when compared to a recent study by Luetkens et al. [11]. They demonstrated a considerably better diagnostic performance of GLS_LV_ alone (AUC 0.83) in their small patient cohort, which might be due to the fact that they did not differentiate patients on the basis of LVEF and thereby introduced LVEF as a biasing factor.

In the present study, GCS_LV_ exhibited a slightly higher diagnostic potential than GLS_LV_, either when used alone or in combination with madSD ± LGE. Nevertheless, the differences between the diagnostic potential of the two LV strain parameters was only small and there might be a role for both parameters in the diagnostic algorithm of suspected acute myocarditis, as already proposed by other groups [11, 16]. However, the better reproducibility of GCS_LV_ as compared to GLS_LV_ [38, 39] as well as the better agreement with tagging derived parameters representing the gold standard for myocardial strain analysis [39] speak in favor of using GCS_LV_ in future diagnostic approaches.

### T2 mapping derived myocardial tissue parameters

Like in the initial proof of concept [17] and the subsequent confirmation study [20], madSD exhibited a high diagnostic potential in the present study cohort and its diagnostic performance was better than for any other tested T2 parameter and even than combined LLC. This and the controversial results of previous studies using averaged myocardial T2 values for diagnosing suspected acute myocarditis [7, 10–13, 18] underline the potential of the novel approach of “mapping tissue inhomogeneity” in acute myocarditis, as myocarditis lesions exhibit a focal nature and often are confined to small areas with abnormal T2 times compared to large areas of myocardial tissue exhibiting normal T2 times [40]. In addition, measuring global T2 times is of limited diagnostic value when it comes to multi-center, multi-vendor studies, as T2 times are known to vary with the sequence and field strengths used [23]. However, it is still unclear whether madSD might be more independent of such technical factors when compared to global T2 values. Further studies therefore should look at the potential of madSD to overcome the sequence and field strengths dependency [23] of mapping techniques.

Interestingly, combining madSD with maxT2 in a model revealed some collinearity between the two parameters in the present study and unlike the results of our previous studies, combining madSD with maxT2 in the present study cohort was not superior to using madSD alone as a predictor. As maxT2 represents a parameter, which might be more prone to outliers e.g. related to artifacts (as a single artifact in one segment has the inherent potential to cause a false-high segmental T2 value) [20], using madSD alone for detecting myocardial inflammation might be the better choice for future diagnostic algorithms.

### Multiparametric imaging models

Although madSD alone already yielded an excellent diagnostic performance in the present study cohort, the proposed multiparametric models exhibited an inherent potential to further improve the diagnosis of suspected acute myocarditis. Using the best multiparametric model in the present study, i.e. the combination of madSD, GCS_LV_ and LGE missed only 2 patients with suspected acute myocarditis (compared to 28 missed by LLC). This parallels the results of Luetkens et al. [11] who recently could demonstrate the incremental diagnostic value of a combination of LGE with global myocardial T2 over LLC or single quantitative CMR parameters.

Even without the use of gadolinium, the combination of madSD and GCS_LV_ exhibited an excellent diagnostic performance in our study cohort, missing only 5 patients with suspected acute myocarditis. This approach might prove especially advantageous in patients with contraindications for the use of gadolinium-based contrast agents. In addition, there is a particular need for a diagnostic tool allowing an identification of LGE-negative subjects with acute myocarditis [11].

We tested the diagnostic performance of different parameters in myocarditis patients compared to a group of healthy subjects. However, the most relevant challenge for the novel quantitative CMR parameters will be their discriminative power in myocarditis patients when compared to patients with other non-inflammatory cardiomyopathies [7] or between patients with acute and chronic forms of myocarditis, as CMR generally appears to provide a better diagnostic performance in the acute setting of myocarditis than in subacute or chronic stages [6, 7, 40, 41]. In addition, we did not include T1 and ECV mapping into our multiparametric models. Yet, two recent studies including T1 and ECV mapping could already demonstrate that only T2, but not T1 or ECV provided an adequate diagnostic performance in patients with acute as well as chronic symptoms and between patients with inflammatory and non-inflammatory dilated cardiomyopathy [7, 12]. Moreover, Luetkens et al. showed an equal diagnostic performance for global myocardial T1 and T2 values in acute myocarditis [11], both parameters being influenced by myocardial water content. Finally, the addition of clinical e.g. serological parameters such as Troponin or markers of inflammation to a multiparametric imaging model might further improve its diagnostic potential. Therefore, future studies will have to address the question whether a combination of T1 and T2 mapping or of other clinical, e.g. serological parameters adds some value to the diagnostic work-up of suspected acute myocarditis. Moreover, further studies should focus on the discriminatory power of madSD between patients with acute and chronic forms of myocarditis or between other types of cardiomyopathy.

### Study limitations

The present study has several limitations. First, there are inherent drawbacks of the retrospective study design. Future larger (and multicenter) studies with a prospective selection of patients with suspected acute myocarditis should be performed in order to validate the presented proof of principle experiment of a novel multiparametric imaging approach. In addition, further studies should also evaluate the discriminatory power of the presented multiparametric imaging approach in order to differentiate between patients with acute and chronic forms of myocarditis as well as between other types of cardiomyopathy.

The present study was performed using a clinical reference standard of myocarditis patients and we did not perform EMB. However, EMB is unnecessary or even contraindicated in myocarditis patients with normal systolic function [42]. Hence, we carefully defined the present patient cohort based on clinical criteria similar to other studies using a clinical reference standard [5, 11, 13, 14, 17]. Nevertheless, some patients in whom a differential cause of disease might have been missed despite careful patient selection may have confounded our study cohort.

The results concerning the novel T2 parameter madSD still must be considered as preliminary. As so far only two studies have investigated the diagnostic potential of madT2 in myocardial inflammation, this needs further validation or re-estimation in larger prospective, multi-center studies.

## Conclusions

A multiparametric imaging model including the novel T2-mapping derived parameter madSD, the feature tracking derived strain parameter GCS_LV_ and LGE yields superior diagnostic sensitivity in suspected acute myocarditis when compared to any imaging parameter alone and to LLC.

## Abbreviations

AIC: Akaike Information Criterion
b-SSFP: Balanced steady-state free precession
CMR: Cardiovascular magnetic resonance
ECG: Electrocardiogram
EGE: Early gadolinium enhancement
EGEr: Early gadolinium enhancement ratio
EMB: Endomyocardial biopsy
FT: Feature tracking
GCS_LV_: LV global circumferential strain
GLS_LV_: LV global longitudinal strain
GraSE: Gradient spin echo technique
LGE: Late gadolinium enhancement
LLC: Lake Louise Criteria
LV: Left ventricle/left ventricular
LVEF: Left ventricular ejection fraction
madSD: Mean absolute deviation (MAD) of segmental pixel-SD
maxT2: The one segment of all 16 segments with highest segmental T2
MLE: Maximum likelihood estimator
ROC: Receiver operating curve
ROIs: Regions of interest
RV: Right ventricle/right ventricular
SAX: Short axis
SD: Standard deviation
SR: Strain rate
T2 BB: T2 black blood
